# The translating bacterial ribosome at 1.55 Å resolution generated by cryo-EM imaging services

**DOI:** 10.1038/s41467-023-36742-3

**Published:** 2023-02-25

**Authors:** Simon A. Fromm, Kate M. O’Connor, Michael Purdy, Pramod R. Bhatt, Gary Loughran, John F. Atkins, Ahmad Jomaa, Simone Mattei

**Affiliations:** 1 EMBL Imaging Centre, European Molecular Biology Laboratory, Meyerhofstraße 1, 69117 Heidelberg, Germany; 2 School of Biochemistry and Cell Biology, University College Cork, Cork, T12 XF62 Ireland; 3 Department of Molecular Physiology and Biological Physics, School of Medicine, University of Virginia, Charlottesville, VA USA; 4 MRC Laboratory of Molecular Biology, Cambridge, CB2 0QH UK; 5 Centre for Cell and Membrane Physiology, University of Virginia, Charlottesville, VA USA; 6 Structural and Computational Biology Unit, European Molecular Biology Laboratory, Meyerhofstraße 1, 69117 Heidelberg, Germany

**Keywords:** Cryoelectron microscopy, Ribosomal proteins, Ribosome

## Abstract

Our understanding of protein synthesis has been conceptualised around the structure and function of the bacterial ribosome. This complex macromolecular machine is the target of important antimicrobial drugs, an integral line of defence against infectious diseases. Here, we describe how open access to cryo-electron microscopy facilities combined with bespoke user support enabled structural determination of the translating ribosome from *Escherichia coli* at 1.55 Å resolution. The obtained structures allow for direct determination of the rRNA sequence to identify ribosome polymorphism sites in the *E. coli* strain used in this study and enable interpretation of the ribosomal active and peripheral sites at unprecedented resolution. This includes scarcely populated chimeric hybrid states of the ribosome engaged in several tRNA translocation steps resolved at ~2 Å resolution. The current map not only improves our understanding of protein synthesis but also allows for more precise structure-based drug design of antibiotics to tackle rising bacterial resistance.

## Abstract (web summary)

Developments in cryo-EM sample preparation and data collection are pivotal for structure determination. Fromm et al. present a 1.55 Å structure of the translating bacterial ribosome that provides new insights on its function and may allow for more precise structure-based drug design.

## Introduction

Cryo-electron microscopy (cryo-EM) allows for detailed structural determination of biomolecules, and it is currently the method of choice for obtaining high-resolution reconstructions of large and conformationally heterogenous macromolecular complexes vitrified in functional buffers after purification or directly within their native cellular context^1–3^. Although a remarkably powerful tool for structural biologists, cryo-EM experiments still rely on access to both high-end instrumentation and advanced expertise in sample preparation, data acquisition and image processing, thus posing major challenges for a growing number of life scientists that approach this method to tackle their biological questions for the first time. To address the ever-increasing demand for access to cryo-EM infrastructure and expertise, several local and national facilities have been established to provide access to cutting-edge transmission electron microscopes and project support by expert staff. To this end, the European Molecular Biology Laboratory (EMBL) has recently established the EMBL Imaging Centre, a new service unit whose main mission is to rapidly make the most advanced microscopy technologies available as a synergistic service portfolio to the international user community while providing comprehensive support from sample preparation to image acquisition and data analysis.

To demonstrate the capabilities of our cutting-edge instrumentation and experienced team to support external user projects requiring cryo-EM single particle analysis workflows, we vitrified, imaged, and analysed purified *E. coli* ribosomes stalled during translation. The bacterial 70 S ribosome is an ideal candidate due to its fundamental importance in protein synthesis^4–7^ as well as being an active antibiotic target for antimicrobial research^8^. Furthermore, capturing intrinsic conformational states of the ribosomes, that usually represent challenges for structural biology approaches, is imperative to gain an atomic level understanding of the function of this intricate machine.

The ribosome has been a suitable target for single particle cryo-EM due to its large size, globular shape, and high RNA content, which generates relatively high contrast in the otherwise noisy cryo-EM images. Despite this, high-resolution structures (<2 Å resolution) reported recently were limited to protein complexes with high symmetry such as apoferritin^9–12^, aldolase^12^ or β-galactosidase^13^. In contrast, the most detailed reconstructions of ribosomal complexes were so far resolved to 2.0 Å and 2.2 Å resolution, for the bacterial and the mammalian ribosomes^14,15^, respectively. This was mostly due to the lack of intrinsic symmetry of the ribosomes and their conformational and compositional heterogeneity, deriving from inter-subunit movements, flexible expansion rRNA segments, and the intermittent interactions with a plethora of additional translational factors involved in the different steps of translation.

Here, we describe how open access to state-of-the-art transmission electron microscopes combined with bespoke user support enabled structural determination of the bacterial translating ribosome at 1.55 Å resolution. The structure, combined with previous insights, resolves many important features of the ribosome at atomic detail including chemical modifications, magnesium and potassium ions, as well as water molecules. The current resolution also allows for direct identification of multiple rRNA variations including a base pair swap in one of the rRNA helices that is distinct between different *E. coli* strains. The cryo-EM structure contained highly resolved features in the periphery of the ribosome including the ribosomal protein bL9, which provides insights on its possible function. Finally, the current dataset allowed us to resolve several conformational states of the ribosome with different tRNAs from a relatively small number of particles (~20,000 particles) at ~2 Å resolution.

## Results

### Isolation of translating ribosomes for cryo-EM structure determination

To isolate bacterial ribosomes actively engaged in translation, we employed a cell free in vitro translation PURE system^16,17^ of a mRNA encoding a FLAG-tagged nascent chain used for purification. The reaction mixture was incubated at 37 ^ο^C to allow the ribosome to engage mRNA and start several elongation cycles, and after 30 min was placed on ice to pause translation. The translating ribosomes were isolated via a FLAG-tag affinity purification step followed by an ultracentrifugation step to pellet ribosomes from the preparation. Purified ribosomes were then applied to cryo-EM grids for imaging. We rationalised that the sample would contain translating ribosomes frozen at different functional states that can be further purified in silico by a combination of 3D image classification and focused 3D refinement approaches.

### Cryo-EM sample screening at the EMBL Imaging Centre

**Fig. 1:**
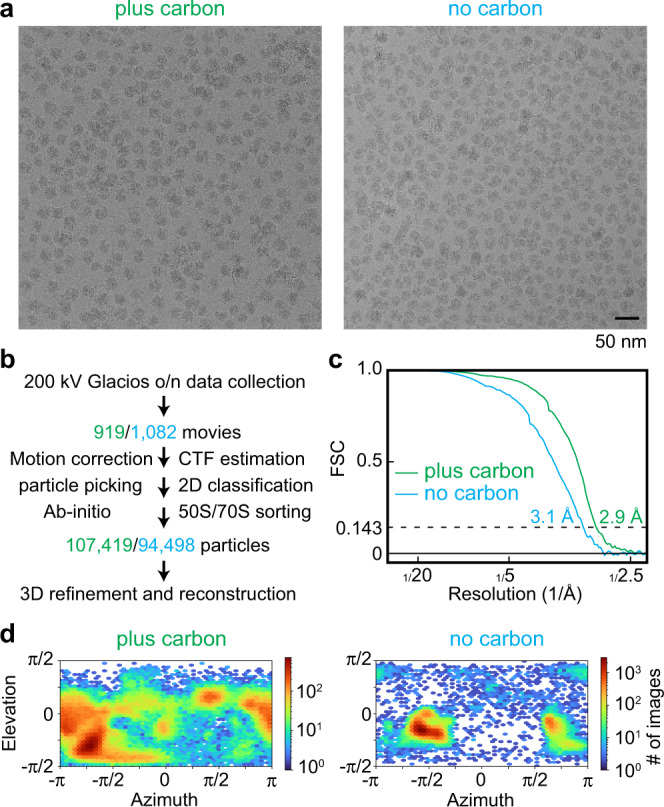
Initial Cryo-EM sample screening to select the grid for high-resolution data collection. **a** Cryo-EM micrographs of the ribosome particles applied to holey carbon grids with (plus carbon, green) and without (no carbon, blue) an additional thin layer of continuous carbon (2 nm). One micrograph out of 919 (plus carbon) or 1082 (no carbon) is shown. **b** Sample screening processing scheme using cryo-EM data collected overnight (o/n) on a ThermoScientific Glacios electron microscope. Colours as in **“a”**. **c** Fourier Shell Correlation (FSC) plots for the data collected on holey grids with and without an additional layer of continuous carbon. **d** Orientation distribution of the particles from data collected on plus and no carbon grids.

To produce and identify a cryo-EM grid suitable for high-end data collection on a Titan Krios microscope, a total of 19 grids were vitrified over three freezing sessions and screened in two sessions using a Glacios (ThermoScientific) transmission electron microscope. At first, the screening was aimed at optimising sample spreading on the grid and ice thickness. Here, two different vitrification devices (ThermoScientific Vitrobot Mark IV and Leica EM GP2) and multiple different grid types (holey carbon grids with and without an additional layer of continuous carbon) were employed. Grid quality assessment was based on the low magnification grid overview which was acquired for each grid (commonly termed “grid map” or “grid atlas”) and high magnification screening images from those grids which showed promising ice thickness and had sufficient imageable areas for potential high-resolution data collection (Fig. 1 and Supplementary Fig. 1a, b).

During the second stage of screening, the selected grids were assessed for ribosome particle density and distribution. A small overnight data set (~1000 movies) was then collected from the most promising grid at the end of each Glacios screening session. The two selected grids were a Quantifoil R2/1 grid with an additional 2 nm layer of continuous carbon vitrified with the Vitrobot (“plus carbon” grid) and a Quantifoil R2/1 grid without the additional layer of continuous carbon vitrified with the EM GP2 using back side blotting to increase particle density in the foil holes (“no carbon” grid) (Fig. 1a and Supplementary Fig. 1b, c). Each dataset was analysed by employing the same processing workflow yielding 3D reconstructions of the 70 S ribosome at ~3 Å resolution from approximately 100,000 particles (Fig. 1b, c and Supplementary Fig. 1d). Despite the similar nominal resolutions obtained, the ‘plus carbon’ dataset had a substantially better particle orientation distribution compared to the “no carbon” dataset which suffered from severe preferred orientation (Fig. 1d). Therefore, we selected the ‘plus carbon’ grid for further high-end data collection on a Titan Krios G4 (ThermoScientific) equipped with a cold-FEG electron source, Selectris X energy filter and Falcon4 direct electron detector; over a 3-day session, a total of ~20,000 movies were collected.

### High-resolution structure of P-site bound translating ribosome

We chose to process particles using a combination of both cryoSPARC^18^ and RELION^19^ software. Particle picking was done semi-automatically with crYOLO^20^ using the pre-trained general model, then followed by standard 2D classification and ab initio model generation which suggested the sample contained both translating ribosomes and free 50 S subunit particles. Heterogeneous refinements in cryoSPARC were then employed to remove particles that belong to the free large ribosomal subunit and resulted in ~750,000 70 S ribosome particles. Further 3D classification resolved a class containing ~650,000 particles of a translating ribosome containing P-site tRNA and a weaker density for the E-site tRNA with the small 30 S subunit in the unrotated state (Supplementary Fig. 2–4). This is a widely observed state for translating ribosomes isolated using in vitro translation systems^21–23^. In this map, the P-site tRNA is tethered to a nascent chain (NC) density with weak density traceable to the backbone of the alpha carbons (Supplementary Fig. 5). However, clear densities of side chains were not observed in the polypeptide exit tunnel due to averaging of several states during translation and thus we did not fit an atomic model in this area.

A 3D variability analysis focused on the tRNA sites identified two subclasses with P- and E-site (~500,000 particles) and A-, P- and E-site (~150,000 particles) tRNA densities, respectively. A combination of 3D refinement schemes, contrast transfer function (CTF) refinement schemes as well as particle polishing^19^ were then used to obtain a final reconstruction to 1.55 Å overall resolution of the larger class containing EM-densities corresponding to P- and E-site tRNAs (Supplementary Fig. 2 and 4, Supplementary Table 1). The local resolution of the small subunit was slightly lower (~1.9 Å) and was further improved to ~1.7 Å after performing focused 3D refinements and signal subtraction of the large subunit (Supplementary Fig. 2).

**Fig. 2:**
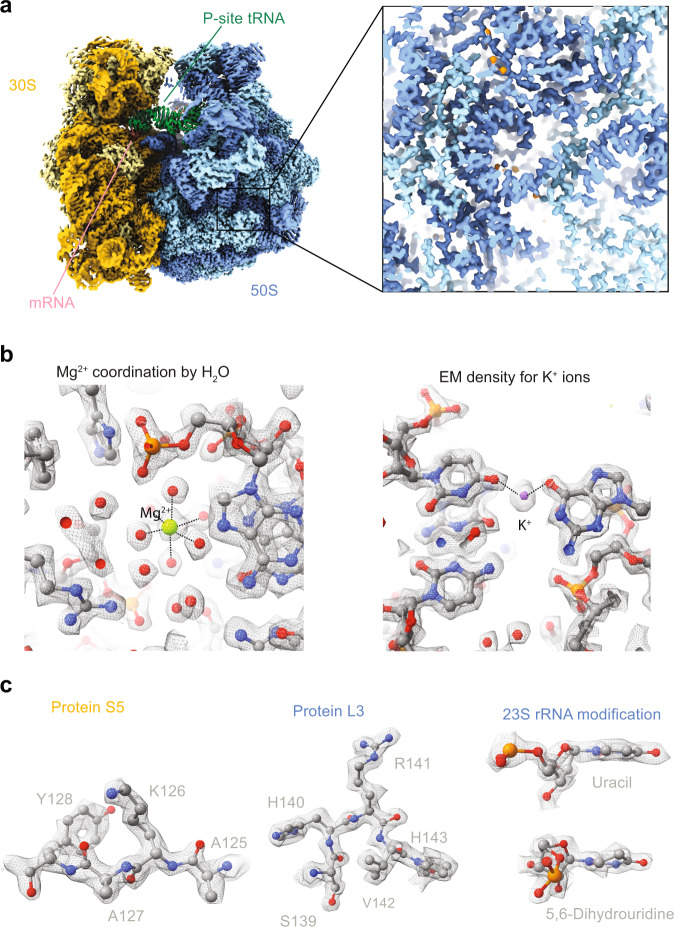
1.55 Å resolution reconstruction of the 70 S ribosome. **a** Cryo-EM map of the bacterial translating ribosome at 1.55 Å coloured in dark yellow (16 S rRNA), light yellow (ribosomal proteins in the small subunit), light blue (large subunit ribosomal proteins), dark blue (23 S and 5 S rRNA). **b**, **c** Closeup densities of protein side chain conformation, water molecules, magnesium, and rRNA modifications are shown as sticks with overlaid EM-densities shown as mesh. Density thresholds: 0.9 (Mg^2+^, **b**) and (K^+^, **b**), 0.5 (Protein S5, **c**), 0.65 (Protein L3, **c**) and 0.8 (23 S rRNA modification).

In the obtained structure, we can visualise rRNA modifications and protein side-chain conformers (Fig. 2) similar to those observed in a previously reported structure of the bacterial ribosome^15^ bound to mRNA and tRNA at 2.0 Å. At the current resolution, we can observe separate EM-densities of water molecules coordinating magnesium ions bound to the RNA backbone (Fig. 2b). In addition, we modelled EM-densities for potassium ions (Fig. 2b), based on their identified positions in a recent X-ray structure^24^. Although the state of the ribosome is paused at different states during translation, we visualised the mRNA with defined anticodon-codon interactions being visible (Supplementary Fig. 6). It is thus likely that the ribosome is paused at preferred sites due to visible densities of purine and pyrimidine rings. However, a reliable assignment of the pausing site could not be assigned and is not further discussed here.

### A C-G base-pair swap in the 23 S rRNA observed in the high-resolution map

**Fig. 3:**
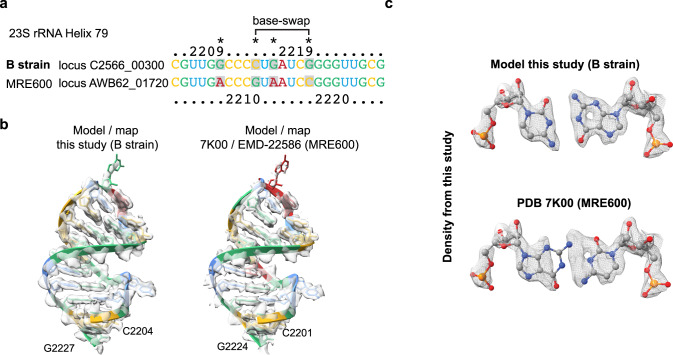
Direct sequencing of rRNA polymorphism resolved by the high-resolution 70 S structure. **a** Partial sequence alignment of H79 of the 23 S rRNA between the *E. coli* B-strain and MRE600. Base-swap positioning is indicated. Other observed mutations are indicated by an asterisk. **b** Snapshot of H79 shown as cartoon illustration with base colouring as in “**a**”. Overlaid cryo-EM density is shown as transparent isosurface. **c** Close-up of the base pair swap using B-strain sequence and MRE600 sequence (PDB 7K00) with overlaid EM density shown as mesh.

Interestingly, we observed a swap between the *E. coli* MRE600 strain sequence of the 23 S rRNA docked into our cryo-EM map. The initially identified swap was a G-C base-pair swap in the position G2210 and C2216 (MRE600 numbering) in helix 79 of the 23 S rRNA. After closer inspection, we realised that the PURE in vitro translation system kit was developed based on the *E. coli* B-strain instead^25,26^, of which the sequence fits well into the EM-density of our maps. Through sequence alignments of the seven different 23 S rRNA operons of the *E. coli* B-strain and comparing their fit to our EM density we could identify loci C2566_01480 and C2566_04790 as the predominant ones in our 70 S ribosome sample. There are two additional mutations within the same rRNA helix which fit to our density (Fig. 3a–c). All other differences between the MRE600 and B-strain rRNA sequences (mutations and insertions), are also reflected by our EM map. Sequence polymorphisms within the 23 S and 16 S rRNA is a common property of bacterial strains and environmental isolates of microbes which could be used as a possible indicator of strain virulence^27,28^. The C-G swap identified here is an intriguing example of co-evolution where two separate mutations in the 23 S rRNA sequence were attained to maintain the base-pairing and the integrity in the 23 S rRNA helix.

### Density corresponding to the ribosome protein bL9

**Fig. 4:**
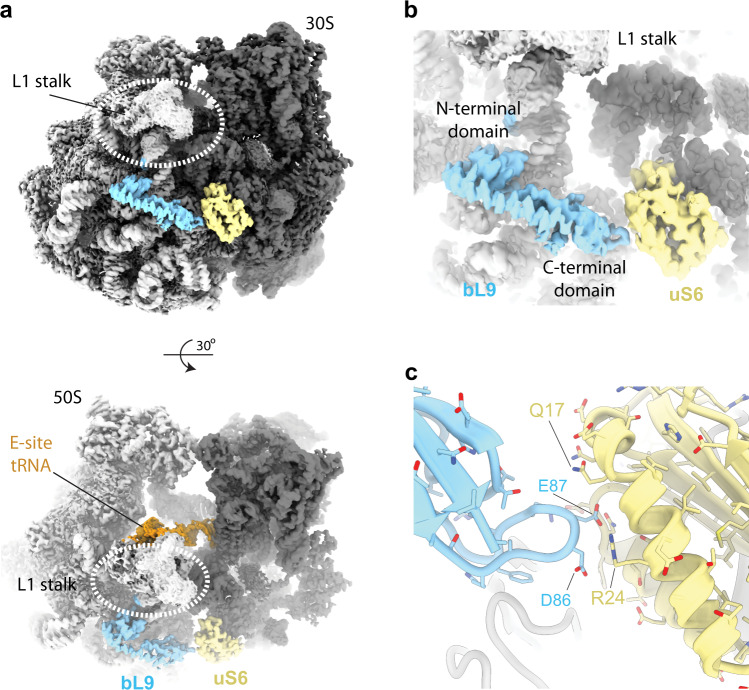
Full-length bL9 in the closed conformation resolved by the high-resolution 70 S structure. **a** bL9 (cyan) and uS6 (yellow) highlighted in the context of the cryo-EM map of the 70 S ribosome. Large subunit is in light grey, small subunit in dark grey. L1 stalk of the ribosome is indicated. **b**, **c** Closeups of bL9 density with surrounding interactions with the small subunit. Ribosomal protein uS6 is indicated. Overlaid EM density is shown as a transparent surface in (**b**). Colouring as in (**a**).

In the current map, the spread of local resolution at the periphery is around 2–3 Å (Supplementary Fig. 7a), which underscores the stability of our sample preparation and data processing schemes. We observe a well-resolved density at side-chain resolution of the ribosome proteins bL9 and uS6 (Fig. 4a–c, and Supplementary Fig. 7b, c). bL9 is located at a peripheral position of the ribosome forming a strut-like shape below the L1 stalk and bridging the large and the small subunits of the ribosome. The C-terminal domain of bL9 is highly mobile and was observed in an open conformation in previous X-ray structures^29^ and later observed in the closed conformation in high-resolution cryo-EM structures^30^ contacting the small ribosomal subunit. In the current structure, bL9 is observed in its entirety with the C-terminal domain establishing contacts with uS6 where it locks the L1 stalk in the closed conformation contacting the E-site tRNA (Fig. 4a). The closed conformation of this protein is stabilised via a salt bridge between Arg24 (uS6) and Glu87 (bL9) (Fig. 4c). Notably, the strut-like shape of bL9 is rich in negatively charged residues through its extended structure (Supplementary Fig. 7d), which would otherwise repel away from the ribosome. Furthermore, a mutant in the C-terminal domain (S93F) affects forward slippage on mRNA, suggesting a role for L9 in fidelity of translation^31^. We speculate that this unusual feature of a ribosomal protein carries an important function during protein synthesis that could also contribute to its detachment and flexibility in previous X-ray and cryo-EM structures that were done under variable salt conditions.

The ribosomal protein bL9 plays an important role in influencing reading frame maintenance and restraining forward slippage on mRNA. This restraint can be circumvented by recoding signals that cause programmed frameshifting that are availed of by many viruses and mobile elements^31–33^. Recently, L9 interactions across neighbouring ribosomes translating the same mRNA were also shown to play a role in polysome formation^34^ and ribosome quality control pathways bridging interaction between two colliding ribosomes^35,36^. In addition, L9 interacts with elongation factor P (EF-P)^37^ as well as the ribosome biogenesis GTPases EngA^38^, extending the current known function of this protein. High-resolution of bL9 is thus highly pertinent to determining the outstanding functional aspects of this enigmatic dual-mode protein.

### Multiple functional states of the translating bacterial ribosome

**Fig. 5:**
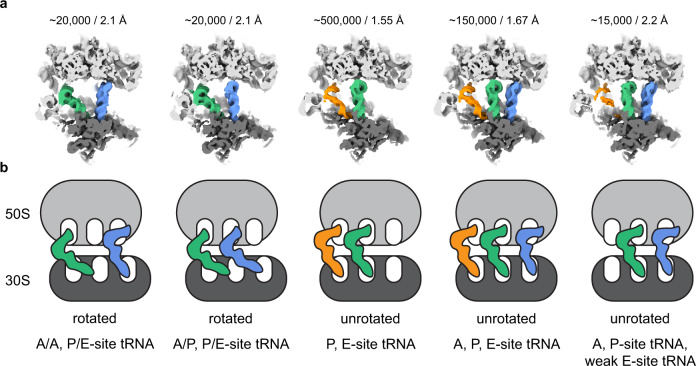
Different tRNA states revealed by 3D classification and variability analysis at high resolution from a small number of particles. **a** Cryo-EM densities of different ribosome translating states from 3D variability analysis low-pass filtered at 5 Å. Large ribosomal subunit coloured in light grey, small subunit in dark grey, A-site tRNA in blue, P-site in green, E-site in orange. Number of particles and resolution of the 3D reconstruction for each class are indicated above. **b** Schematic representation of all identified tRNA states in the data set. Colouring as in “a”. The respective tRNA state is indicated below.

We also investigated the presence of additional functional states of the translating ribosome in the dataset. We employed 3D classification to identify a class of ribosomes in the rotated state which was further sub-classified using the 3D variability approach implemented in cryoSPARC by focusing on the tRNA binding sites on the ribosome (Fig. 5, Supplementary Fig. 2 and 3). This classification scheme resolved three tRNA translocation intermediates of the translating ribosomes. Besides the above described classical unrotated state with bound A-, P-, and E-site tRNAs, we also resolved two additional states containing chimeric state rotated ribosomes. The first class is of A/P- and P/E- tRNA hybrid state ribosomes and the second class with A- and P/E-site tRNAs (Fig. 5). The chimeric state was observed in the absence of the tRNA-bound elongation factor EF-Tu and was described in more detail in recent cryo-EM structures^39–41^. Interestingly, each of the two intermediates represent only ~2.7 % (~20, 000 particles) of the total dataset and yet could be refined to ~2.1 Å indicating the possibility to sort for rare intermediates that can be resolved to high resolution from a relatively small number of particles.

## Discussion

In summary, we present multiple structures of the *E. coli* translating ribosome, with the most populated state resolved at a global resolution of 1.55 Å. To our knowledge, this is currently the highest resolution reported for the ribosome or any non-symmetrical complex by single particle cryo-EM. Determining structures of the ribosome has continuously progressed over the last 25 years since the initial X-ray structures emerged^29,42–45^. More recently, cryo-EM has provided insight into a plethora of functional states describing how ribosomes function, some of which were de-convoluted from the same reaction at high resolution. With current developments in cryo-EM image processing, direct pull-downs of active complexes are becoming the go-to approach requirement for resolving short-lived intermediates.

We suggest that a combination of improvements in sample preparation, microscopy hardware, data collection, and processing schemes with respect to previous studies allowed for this substantial advancement of the achievable resolution for an asymmetric and compositionally heterogeneous complex like the ribosome. The highly resolved rRNA in the presented structure allowed for sequence identification of portions of the rRNA directly from the map, where we detected a C-G base pair swap in H79 of the large subunit rRNA. This swap is due to sequence variation in the *E. coli* B-strain, compared to the MRE600 strain. Interestingly, despite the mutations being in two distant positions, the base pair swap does not alter the structural integrity of the helix. Such possible identification of base pair swapping in the rRNA could be used to identify bacterial strains by directly performing sequence analysis on their rRNA using cryo-EM performed on translating ribosomes purified from complex bacterial populations. Finally, the current dataset demonstrates how the latest generation of microscopy hardware and software allowed for the 3D classification and refinement of multiple conformational states of asymmetric complexes from a relatively small number of particles (~20,000) to around ~2 Å. The results presented here show the importance of imaging facilities that provide open access to state-of-the-art instrumentation and tailored support to enable advanced structural studies pursued by researchers interested in cryo-EM as a new method.

## Methods

### Sample preparation

A synthetic DNA sequence (gBlock, IDT) with a T7 promoter, a leader region, and a Shine Dalgarno sequence upstream of sequence encoding an N-terminal 3x FLAG-tag followed by a 96 aa linker and sequence (373 nt) from bacteriophage MGS13 was used as template for PCR using primers gBlock S and gBlock AS (see Supplementary Table 2). PCR amplicons were verified by agarose gel electrophoresis and purified using the Monarch PCR and DNA Cleanup Kit (New England Biolabs). In vitro transcription was performed using the T7 RiboMAX Express Large Scale RNA Production System (Promega), followed by Lithium Chloride precipitation to purify the synthesized RNA.

In vitro translation was carried out using the deltaRF123 Kit (New England Biolabs). 200 ng of mRNA was added per µL of reaction, with a total reaction volume of 1.5 mL. Reactions were incubated at 37 °C for 30 min. Translation reactions were chilled on ice for 10 min to halt translation, and HEPES KOH pH 7.4 was added to a final concentration of 50 mM. Chilled translation reactions were directly incubated with anti-FLAG M2 beads (Sigma) for 2 h at 4 °C with gentle mixing.

The beads were then washed with 50 mM HEPES, pH 7.4, 100 mM KOAc, 25 mM Mg(OAc)_2_, 0.1% Triton X-100, 1 mM DTT; 50 mM HEPES, pH 7.4, 250 mM KOAc, 25 mM Mg(OAc)_2_, 0.5% Triton X-100, 1 mM DTT and RNC buffer (50 mM HEPES, pH 7.4, 100 mM KOAc, 25 mM Mg(OAc)_2_, 1 mM DTT). RNCs were eluted after 3 sequential 10 min incubations at room temperature in the RNC buffer that contained 0.2 mg/mL 3x FLAG peptide (Sigma). The elutions were combined and centrifuged at 186,000 × *g* at 4 °C for 1.5 h in a (TLA-120.1) rotor (Beckman Coulter). Supernatant was discarded and the pellet was resuspended in the RNC buffer at a concentration of ~200 nM. At each step of translation reactions and purification, aliquots were taken to perform SDS-PAGE on 4–12% Bolt Polyacrylamide gels (Invitrogen) and Western blots. Gene products of interest were probed using a commercial anti-FLAG antibody (Sigma Cat. No. F1804).

### Cryo-EM sample preparation

Cryo-EM grids have been prepared with a Vitrobot Mark IV (plus carbon sample, ThermoScientific) or an EM GP2 (no carbon sample, Leica) plunger. Environmental chambers were set to 6 °C and 100% humidity. Grids were rendered hydrophilic by plasma cleaning for 30 s in a 90/10% Ar/O mixture with a Fischione 1070 plasma cleaner right before plunge freezing. 2.5 µL of the undiluted ribosome sample were applied to the foil side of the grid. The plus carbon sample (Quantifoil Cu300 R2/1 + 2 nm C, as commercially available) was blotted for 2 s with blot force +3 after a 30 s wait time after sample application and immediately plunge frozen in liquid ethane. The no carbon sample (Quantifoil Cu200 R2/1) was blotted for 1.5 s from the back side right after sample application and immediately plunge frozen in liquid ethane. In all cases Whatman 597 blotting paper was used.

### Cryo-EM data collection

Grid screening and screening data acquisition was performed on a Glacios TEM operated at 200 kV equipped with a Selectris X energy filter and Falcon 4 direct electron detector (ThermoScientific). Data was acquired at a nominal magnification of 100 kx resulting in a calibrated pixel size of 1.154 Å on the camera. A 50 µm C2 and a 100 µm objective aperture were inserted and the width of the energy filter slit was set to 10 eV. The TEM was operated in nanoprobe mode at spot size 6. Movies were acquired over 8.3 s exposure time with a total accumulated dose of ~40 e^-^/Å^2^ (~6.4 e^-^/px/s over an empty area on the camera level) in counting mode and saved in the EER file format. For the plus carbon data set, 919 movies were acquired in one overnight data collection (1 exposure per hole/stage movement) and for the no carbon data set 1,082 movies were acquired in the same way.

After evaluation of the two screening data sets (see below), the plus carbon grid was selected for high-resolution data acquisition on a Titan Krios TEM operated at 300 kV equipped with a C-FEG, Selectris X energy filter and Falcon 4 direct electron detector (ThermoScientific). A total of 19,449 movies were collected over three days at a nominal magnification of 165 kx resulting in a calibrated pixel size of 0.731 Å on the camera. A 50 µm C2 and a 100 µm objective aperture were inserted and the slit width of the energy filter was set to 10 eV. The TEM was operated in nanoprobe mode at spot size 5 and a beam diameter of ~460 nm. Movies were acquired over a 5.2 s exposure time with a total accumulated dose of ~40 e^-^/Å^2^ (~4.1 e^-^/px/s over an empty area on the camera level) in counting mode and saved in the EER file format (1293 fractions per movie). The target defocus was set to −0.5 to −1.5 µm with 0.1 µm steps between holes. In total, 16 movies were acquired per hole using beam image shift (11 in the outer ring, 5 in the inner ring) before moving to the next hole by stage shift. All TEM screening and data acquisition was performed using SerialEM^46^.

### Data processing and model building

Cryo-EM data processing workflows for the Glacios screening data sets and the Krios high-resolution data set are shown in Fig. 1 and Supplementary Fig. 1, 2 and 3, respectively. Motion correction of EER movies was performed with the CPU implementation of MotionCor2 from Relion4^47^. Initial CTF estimation was performed using CTFFIND4^48^. Gautomatch (https://www2.mrc-lmb.cam.ac.uk/download/gautomatch-056/) was used for particle picking of the Glacios screening data, crYOLO^20^ with the pre-trained general model was used for picking of the high-resolution Krios data set. Particle extraction, 3D classification with alignment as well as Bayesian polishing were performed with Relion4. All other cryoEM data processing steps were performed in cryoSPARC^18^. Separation of 50 S and 70 S particles was achieved via Heterogeneous Refinement, different tRNA states were identified using a combination of 3D classification and 3D Variability analysis. Maps were post-processed by local filtering after local resolution estimation as implemented in cryoSPARC. Conversion of cryoSPARC ‘.cs’ files to Relion ‘.star’ files was performed using UCSF pyem^49^ in combination with in-house written bash scripts^50^ (https://github.com/simonfromm/miscEM). All final Homogeneous Refinements were done with per-particle defocus and per-group CTF parameter optimization as well as Ewald Sphere Correction switched on. Exposures from all beam image shift positions of the acquisition pattern for the dataset collected on the Titan Krios were put into a separate group resulting in a total of 16 exposure/optics groups.

Model building was started with individually rigid-body fitting all chains from the published 2 Å *E. coli* 70 S ribosome structure (PDB 7K00) in COOT^51^. After manual inspection of the rRNA of the large and small ribosomal subunits for mutations, the residue numbering of the *E. coli* B-strain was used. All chains were inspected and adjusted manually in COOT and subsequently refined with real-space refinement in PHENIX^52^ against the unsharpened map with secondary structure and Ramachandran restraints switched off (Supplementary Table 1). The C-terminal part of bL9 absent from PDB 7K00 was built de novo based on the AlphaFold2 model. Water molecules were added to the 30 S and 50 S part of the structure separately using “Find Waters” in COOT. Mg^2+^ and K^+^ positions were inferred from PDB 7K00 and 6QNR, respectively. All Mg^2+^ and K^+^ ion positions were checked and modified manually when necessary. The model geometry was validated using MolProbity^53^. The model vs. map FSC at FSC = 0.5 coincides well with the one determined between the map half-sets at FSC = 0.143 (Supplementary Fig. 4). All cryoEM density and model renderings were generated with ChimeraX^54^.

### Reporting summary

Further information on research design is available in the Nature Portfolio Reporting Summary linked to this article.

## Supplementary information

Supplementary Information

Reporting Summary

## Data Availability

The maps and models generated in this study have been deposited in the EM and protein database under accession codes EMD-15793 (high-resolution structure), EMD-15794 (rotated, A/A an P/E tRNAs), EMD-15795 (rotated, A/P and P/E tRNAs), EMD-15796 (unrotated, A and P tRNAs), EMD-15797 (unrotated, A, P and E tRNAs) and PDB 8B0X (high-resolution structure). All micrographs and coordinates for the picked particles have been deposited in Electron Microscopy Public Image Archive under accession code EMPIAR-11377.
