# Supplementary material for: The translating bacterial ribosome at 1.55 Å resolution generated by cryo-EM imaging services
Source: Nat Commun. 2023 Feb 25;14:1095. doi: 10.1038/s41467-023-36742-3 (PMC9968351; doi:10.1038/s41467-023-36742-3)
Supplement: Supplementary file 1 — Supplementary Information [file 41467_2023_36742_MOESM1_ESM.pdf]

Supplementary Information

The translating bacterial ribosome at 1.55 Å resolution generated by cryo-EM imaging services

Simon A. Fromm, Kate M. O'Connor, Michael Purdy, Pramod R. Bhatt, Gary Loughran, John F. Atkins, Ahmad Jomaa, Simone Mattei

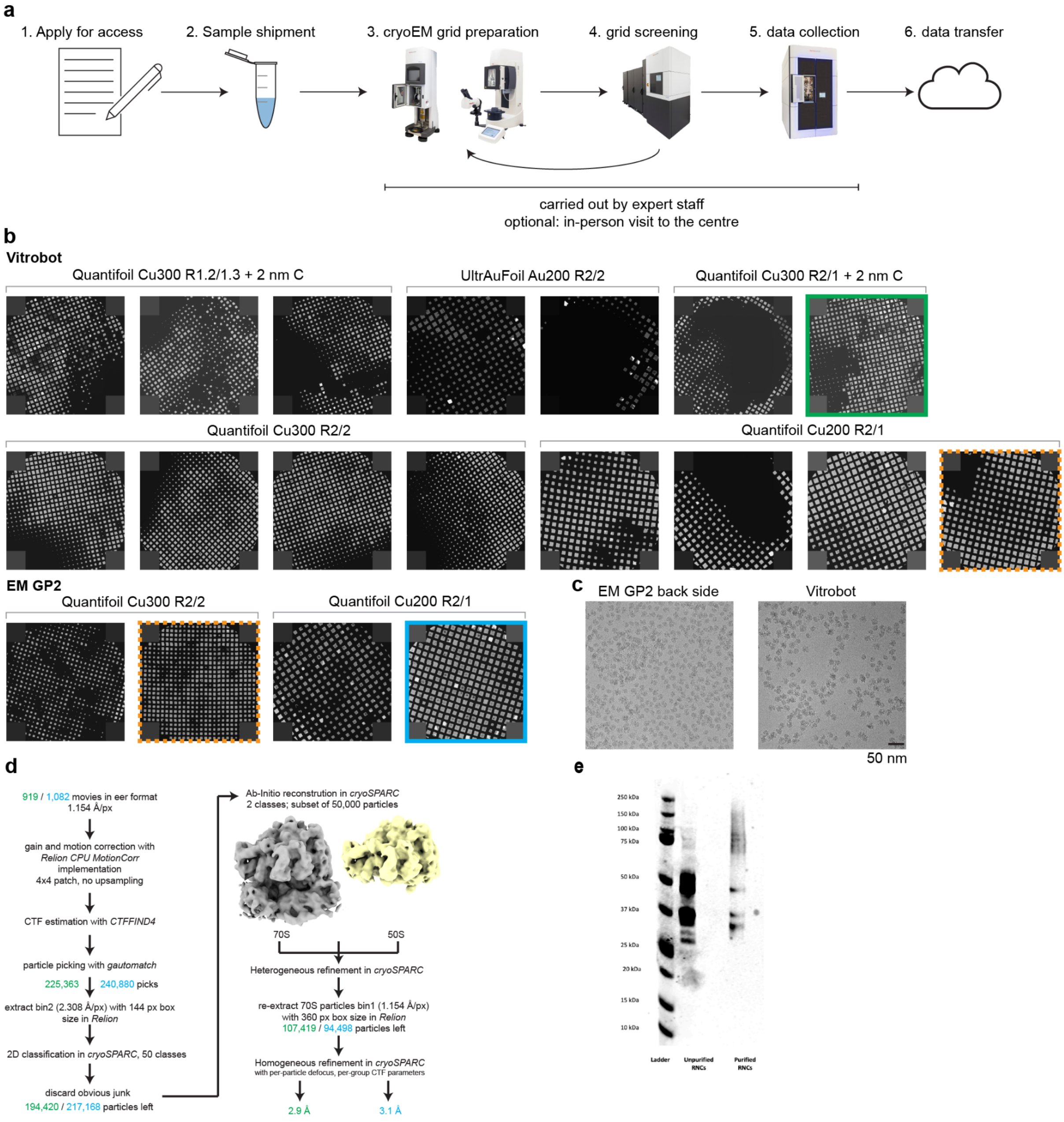

Supplementary Fig. 1 General access scheme/workflow and Ribosome sample screening workflow. a, Schematic of the workflow for cryo-EM sample preparation and data collection at the EMBL Imaging Centre; instruments image credit – Stuart Ingham/EMBL. **b,** Grid maps of all 19 grids prepared from the ribosome sample. Grid type and freezing device are indicated above the respective grid map. The 'plus carbon' grid selected for a Glacios screening data set as well as for the high-resolution Krios data set is boxed in green. The 'no carbon' grid selected for the second Glacios screening data set is highlighted with a blue box. **c,** Representative high-magnification images from 'no carbon' grids highlighted with an orange dashed box in 'b' that were prepared with the EM GP2 (left) and Vitrobot (right). Higher particle density is observed in the grids prepared with the EM GP2 as a result of back-side blotting. **d,** Data processing workflow of the 'plus carbon' (green) and 'no carbon' (blue) screening data sets from the Glacios. **e,** Western Blot of the RNCs, nascent chains were detected using anti-FLAG antibody.

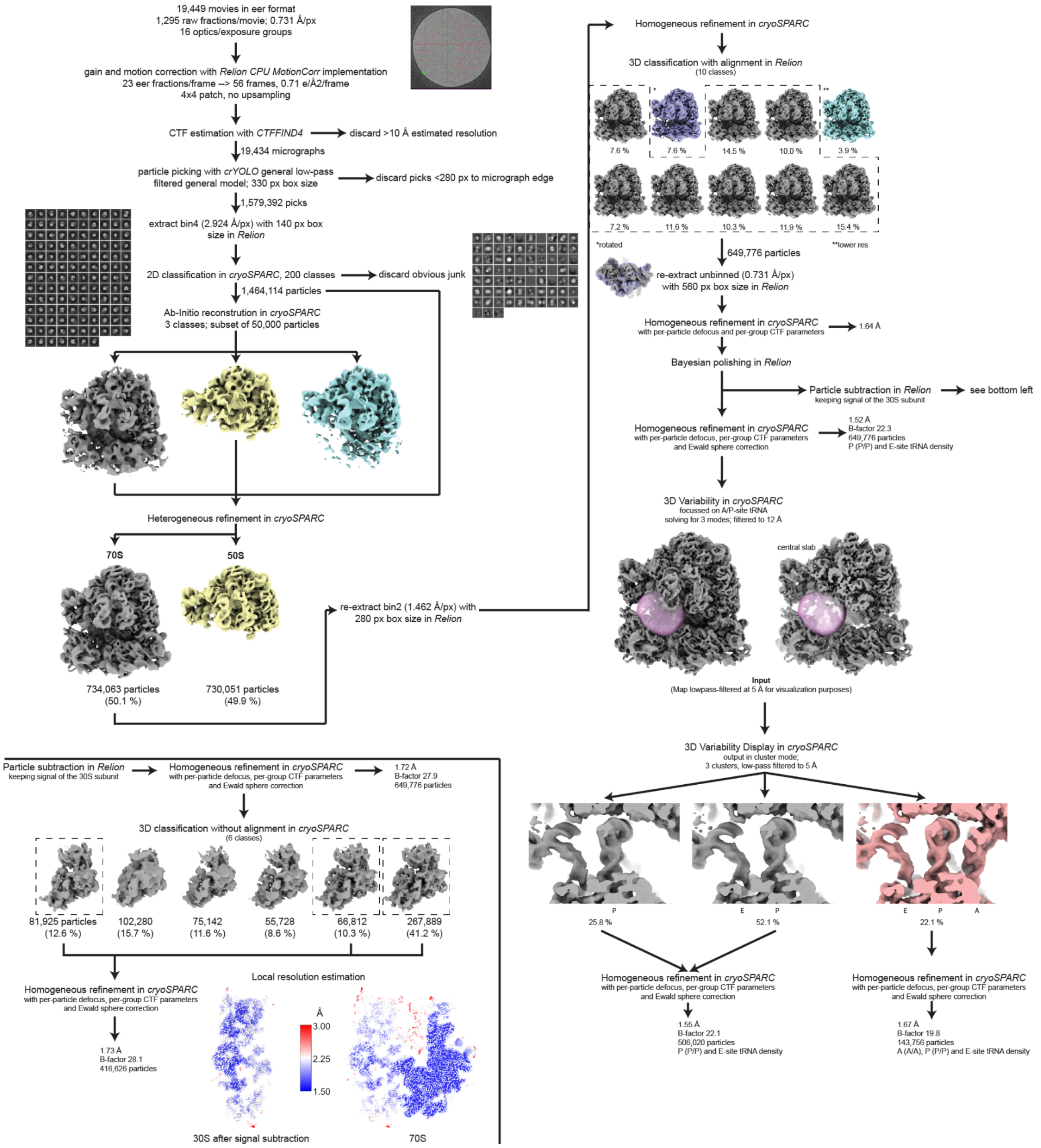

Supplementary Fig. 2 Processing scheme of the ribosome in the unrotated state. Processing scheme to initially separate 70S ribosomes from free 50S subunits by heterogeneous refinement in cryoSPARC. 70S ribosomes were then classified using 3D classification in RELION, which separated ribosomes based on the rotated and unrotated states of the small ribosomal subunit. Ribosomes in the unrotated state were then classified using a binary mask around the tRNA binding site to resolve additional states of bound tRNA. To increase the resolution in the 30S subunit signal subtraction of the 50S part in RELION followed by 3D classification without refinement in cryoSPARC were performed. Note the improved local resolution in the signal subtracted 30S subunit compared to the 70S ribosome. 50S subunits were not further processed.

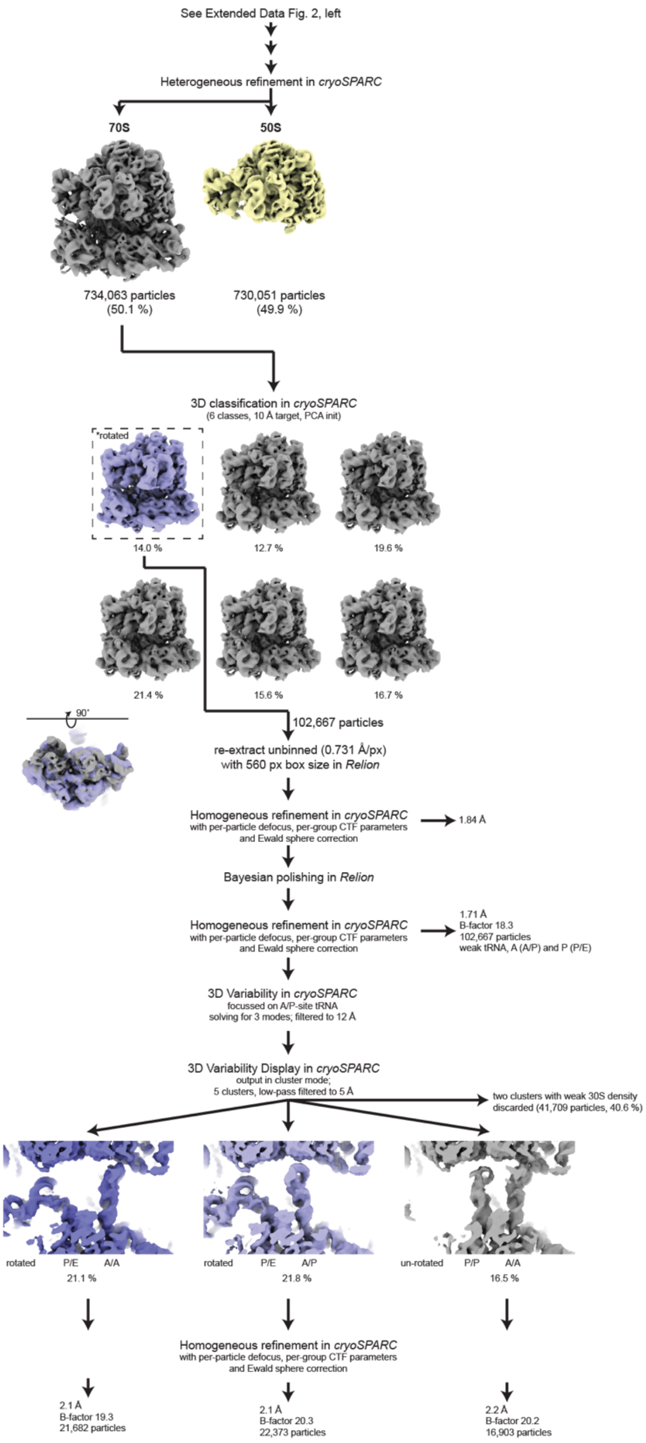

Supplementary Fig. 3 Processing scheme for the ribosome in the rotated state. Ribosomes in the rotated state were classified using a binary mask around the tRNA binding site to resolve additional states of bound tRNA, similarly as described in Supplementary Fig. 2.

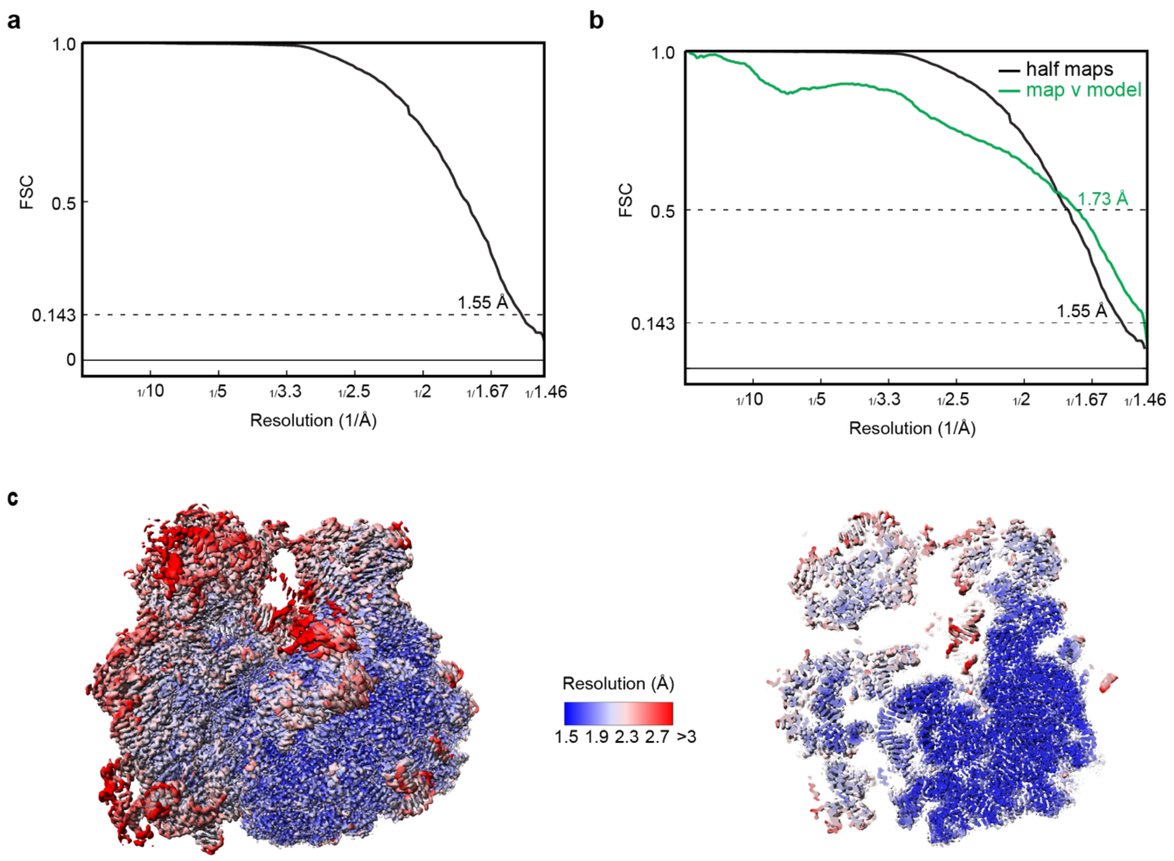

Supplementary Fig. 4 FSC and Local resolution plot of the high-resolution E. coli translating ribosome. a, Gold-standard FSC of final 3D reconstruction**. b**, Map-model validation: gold-standard FSC (black) overlaid with map-vs-model FSC (green). **c**, Local resolution estimation. Left, surface representation of the full ribosome. Right, central slice through the density.

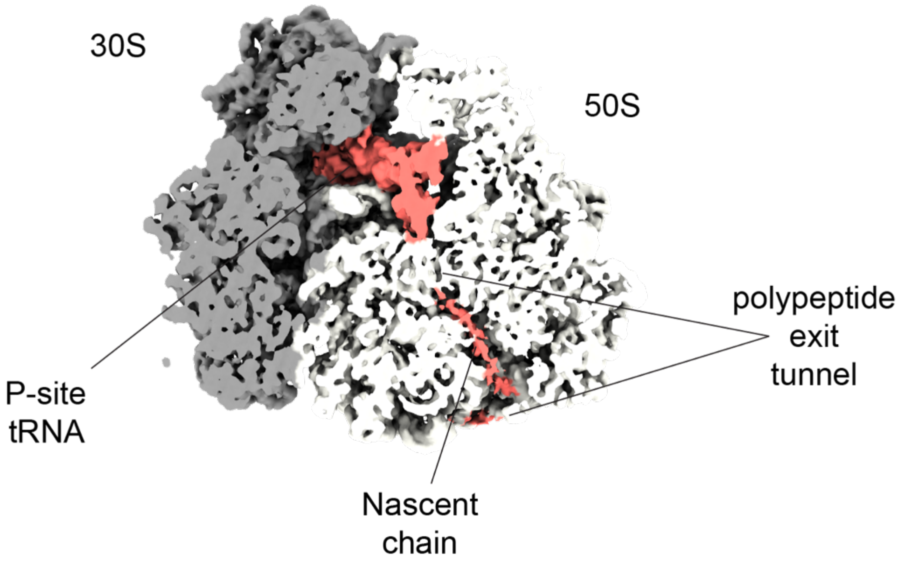

Supplementary Fig. 5 Visualisation of the tRNA-tethered nascent chain in the ribosomal polypeptide exit tunnel. Cross-section of the polypeptide exit tunnel with resolved EM-density for the nascent chain. Ribosomal subunits 30S and 60S are coloured light and dark grey to emphasise the density of the nascent chain shown in red. Cryo-EM density is shown as surface and low-pass filtered to 5 Å resolution.

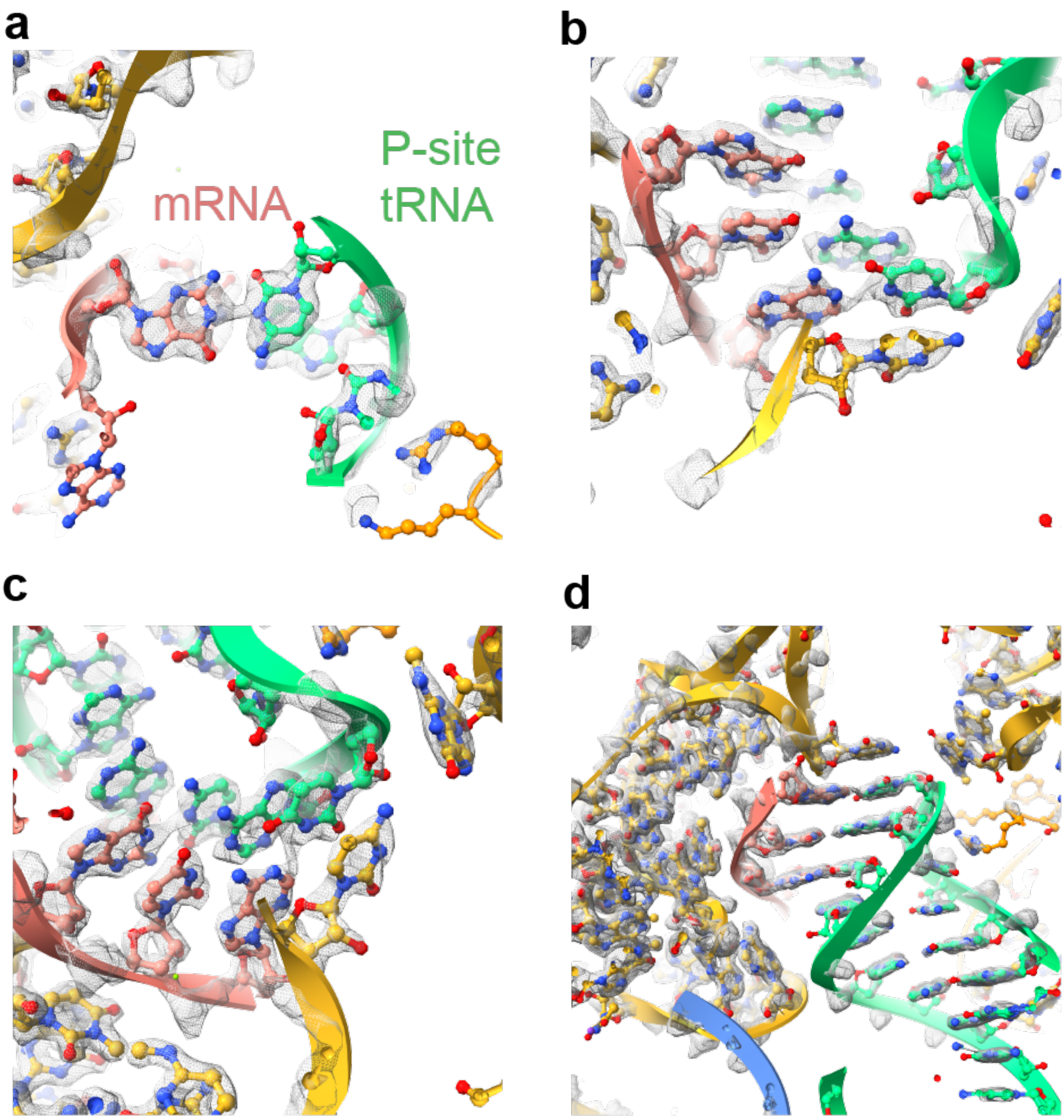

Supplementary Fig. 6 Cryo-EM density corresponding to the codon anti-codon base pairing in the decoding centre of the ribosome. a-d, Close-ups of the codon-anti codon interactions in the translating ribosome with underlying EM-density shown as mesh. Atomic coordinates are shown as sticks and cartoon illustrations and coloured as in main figure 2.

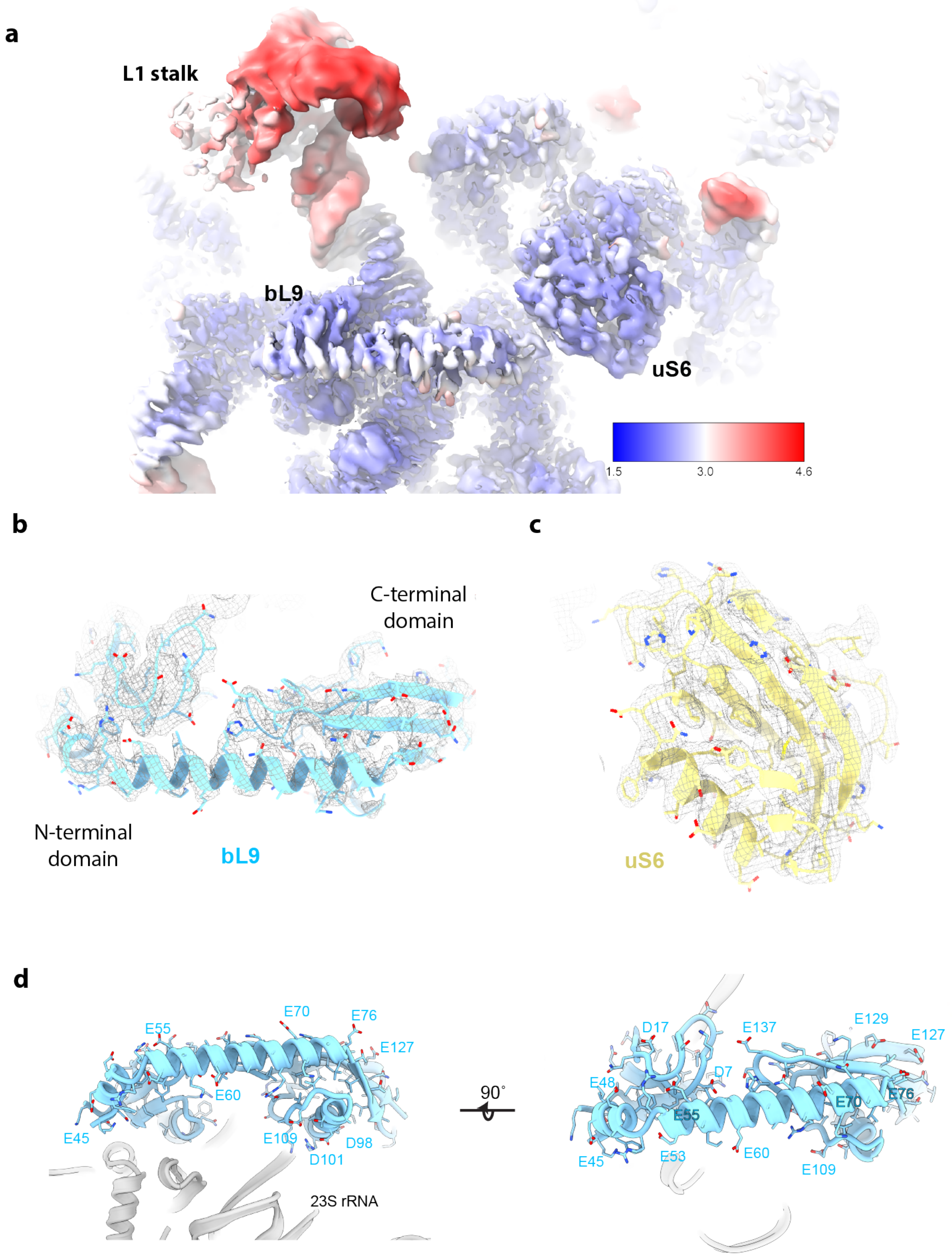

Supplementary Fig. 7 bL9 is rich in negatively charged residues that could repel rRNA. **a,** Local Resolution plot of the L1 stalk, bL9 and uS6 region of the ribosome. **b** and **c,** Coordinates of the ribosomal protein uS6 and bL9 with underlying EM-density shown as mesh. **d**, Close-ups of the ribosomal protein bL9 shown as a cartoon illustration. Sequence shows high distribution of negatively charged residues. Colouring is the same as in main figures.

Supplementary Table 1. Cryo-EM validation and Model Refinements

	**70S ribosome**
EMDB code	EMD-15793 [https://www.ebi.ac.uk/emdb/entry/EMD-15793]
PDB code	8B0X [http://doi.org/10.2210/pdb8B0X/pdb]
**Data collection and processing**	
Magnification	165,000x (nominal)
Voltage (kV)	300
Electron exposure ($e^{-}/Å^2$)	40
Defocus range (μm)	0.5-1.5
Calibrated Pixel size (Å)	0.731
Initial particle images (no.)	1,579,392
Final particle images (no.)	506,020
Map resolution at FSC=0.143 (Å)	1.55
**Structure refinement in PHENIX 1.20.1**	
Model resolution at FSC=0.5 (Å)	1.73
CC_{mask}	0.94
Map sharpening B factor ($Å^2$)	– 22.1
Model composition	
Non-hydrogen atoms	151,543
Protein residues	5,621
RNA residues	4,448
Ligands: $H_2O/Mg^{2+}/K^{+}$	12,027
B factors min/max/mean ($Å^2$)	
Protein	10.42/120.27/56.09
RNA	8.70/184.27/49.53
Ligand	17.89/85.60/40.28
R.m.s. deviations	
Bond lengths (Å)	0.007
Bond angles (°)	0.703
**Validation**	
MolProbity score	1.56
Clashscore	4.12
Poor rotamers (%)	1.50
Protein	
Ramachandran plot	
Favored (%)	96.46
Allowed (%)	3.34
Disallowed (%)	0.20

Supplementary Table 2. Primers and synthetic DNA sequence for programming ribosomes

Primer Name	Primer Sequence
gBlock S	gctcttctaaagaccttaccctacttctcg
gBlock AS	atcctctatgactattaccaggc

Sequence of synthetic DNA used to isolate RNCs:

Gctcttctaaagaccttaccctacttctcgccgtaatacgactcactataggcaacaacaacaaccgt
tagcttccgacacaaggcttttcactagcaactaaggaggtccaccatggcctacaaagaccacgacg
gtgattataaagatcacgacatcgattacaaggacgacgacgacaagtccaaagagccgttgcgcccc
cgttgtcgtcctattaatgccacccttgcggtcgagaaagagggctgtccggtttgcatcacggtcaa
cactactatttgtgctggatattgccctacggcgacccgtgtccttcaggggtcttgcctgctcttc
cccaggttgtatgcaattaccgccgtagtgttactcaccgtatccttactgtccccatcgcgcaggat
caagttggcgcgtattatcagcaacctggtcaacaaaacgcaacctggattgttccgccagggaagct
gtacaaagctagcgatcaccacctcgggatcagcggcggaaaggtagatatgaacgcctaccaacaac
agactaatgtaagaagaaccatgaactgcaaccaggtagatagaaccagcaactttgacaaaatcatc
atcgctacagatgctgatggtgatggttagttggggagtaactggttcacatatcactggtctttaca
tcggtctttttacaagcacttcaaagaaatcctgaagaacaacatctacaagcttcagaccccgatt
gtcacgctgaaagattctaaaggaaagatcaagaattcccaactgcacgtgcaggtcgacggtgtttt
tgcaggacatcatcatcatcatcattaggcaccaccaccaccaccactaggcatcatcatcatcatca
ttatgtaccatcgatacgcgttcgaagcttgcggccgcacagctgtatacacgtgcaagccagccaga
actcgctcctgaagacccagaggatctcgagcaccaccaccaccaccactaatgttaattatgttggg
cgttcctacgctgataaaacagaatttgcctggtaatagtcatagaggat

Supplementary Table 3. Mutations and insertions between B and MRE600 23S rRNA

Numbering B strain	Numbering MRE600 strain	Change (B vs MR600)
358	358	A - G
359	359	U - C
543	543	C - A
545	545	C - G
546	546	U - C
549	549	U - A
550	550	A - G
551	551	U - G
552	552	G - C
554	554	G - U
1173	1173	G - A
1175	-	U insertion
1177	1176	A - G
1180	1179	C - U
1222	1221	G - A
1231	1230	C - U
1520	1519	C - U
1732	1731	C - U
1867	1866	U - C
1872-1873	-	GU insertion
1874	1871	A - C
1876	1873	C - A
2209	2206	G - A
2213	2210	C - G
2215	2212	G - A
2219	2216	G - C
2716	2713	C - U
2801	2798	U - C
